# The impact of IL-17A inhibitors on scalp and gut microbiota in psoriasis

**DOI:** 10.3389/fcimb.2025.1623003

**Published:** 2025-10-06

**Authors:** Wenxia Huang, Yuanyuan Geng, Jie Gong, Weiwei Wu

**Affiliations:** ^1^ Affiliated Dermatology Hospital of Hainan Medical University, The Fifth People’s Hospital of Hainan Province, Haikou, Hainan, China; ^2^ National Key Laboratory of Intelligent Tracking and Forecasting for Infectious Diseases, National Institute for Communicable Disease Control and Prevention, Chinese Center for Disease Control and Prevention, Beijing, China

**Keywords:** psoriasis, microbiota, IL-17A, 16S rRNA, scalp, gut

## Abstract

Objective To investigate the differences in scalp and gut microbial diversity, community structure, and specific microbial species in patients with psoriasis vulgaris before and after treatment with interleukin (IL)-17A inhibitors, compared to healthy individuals. Additionally, the preliminary impact of IL-17A inhibitors on scalp and gut microecology was explored. Methods This study utilized 16S rRNA gene sequencing to comparatively analyze the dynamic changes in scalp and gut microbiota diversity and community composition in patients with moderate-to-severe psoriasis vulgaris before and after treatment with IL-17A inhibitors. The study included 15 patients with a Psoriasis Area and Severity Index score of ≥10 and a sex- and age-matched healthy control group. Scalp scale and fecal samples were collected at three-time points: pre-treatment (baseline), 4 weeks post-treatment, and 12 weeks post-treatment. Results IL-17A inhibitors demonstrated favorable efficacy in treating plaque psoriasis. Following treatment, no statistically significant difference was observed in the alpha and beta diversity of the scalp microbiome between patients with psoriasis and healthy controls. Notably, the abundance of harmful bacteria (*Pseudomonas* species) decreased on the scalp, while beneficial *Bifidobacterium* levels increased. Regarding gut microbiota, significant differences in α-diversity richness were observed compared to healthy controls (P<0.05). Moreover, the abundance of *Roseburia*, *Megamonas*, and the phylum *Bacteroidota* increased, although the Firmicutes/Bacteroidota (F/B) ratio showed no significant change. Conclusion: IL-17A inhibitor therapy has the potential to improve the structure and diversity of the scalp microbiome, gradually restoring it toward a healthier state while also enhancing gut microbiota diversity. These therapeutic effects may be mediated through immune regulation, such as the Th17 pathway modulation, and microbial metabolites like short-chain fatty acids.

## 1 Introduction

Psoriasis vulgaris, the most common form of psoriasis, is a chronic and relapsing inflammatory skin disease that severely affects patients’ quality of life and mental well-being (Psoriasis Committee, Dermatology and Venereology Branch of Chinese Medical Association, 2023). In China, the prevalence of psoriasis is approximately 0.47% (Ding et al., 2012), whereas in some developed countries in Europe and America, it ranges between 2% and 3% (Griffiths et al., 2017). The pathogenesis of psoriasis involves a complex interplay of multiple factors, with genetics playing a crucial role. Genome-wide association studies have identified several susceptibility genes linked to psoriasis, including interleukin (IL)-23 receptor, IL-12B, and tumor necrosis factor-alpha (TNF-α)-induced protein 3. These genes are primarily involved in immune system regulation, particularly within the T helper 17 (Th17) cell signaling pathway. Th17 cells are essential in mediating immune and inflammatory responses. Activated Th17 cells, along with the cytokines they secrete, such as IL-17 and IL-22, play a central role in driving keratinocyte proliferation and skin inflammation (Babaie et al., 2022). Various environmental factors, including infections (e.g., streptococcal infections), psychological stress, smoking, alcohol use, and certain medications, can trigger or exacerbate psoriasis (Xie et al., 2021).

Scalp involvement in psoriasis has drawn significant interest from researchers due to its high prevalence, affecting approximately 80% of individuals with the condition, and its adverse impacts on patients’ psychological well-being and social interactions (Merola et al., 2018; Svedbom et al., 2021). Persistent pruritus and severe scaling of scalp lesions typically lead to a marked reduction in the quality of life of affected patients (Farber and Nall, 1992; van de Kerkhof and Franssen, 2001). It can be the sole symptom of psoriasis or may occur alongside lesions in other parts of the body. Along with palmoplantar psoriasis, genital psoriasis, and nail psoriasis, it is classified as one of the “treatment-resistant” manifestations of psoriasis (Nicolescu et al., 2022; Lupulescu et al., 2025). Furthermore, studies suggest that the presence of scalp psoriasis lesions may indicate a higher risk of developing psoriatic arthritis in the future (Rouzaud et al., 2014).Studies exploring the correlation between cytokines and pruritus have revealed that IL-17-related genes exhibit higher activity in the scalp compared to other psoriatic skin areas (Brembilla et al., 2018). Moreover, patients with psoriasis exhibit elevated transcription levels of the IL-23 gene in their skin compared to healthy controls (Nattkemper et al., 2018). Elevated levels of TNF-α, interleukin-17 (IL-17), and IL-23 are believed to be associated with the upregulation of cyclic adenosine monophosphate, mediated by phosphodiesterase-4 (Sola-Ortigosa et al., 2012). In recent years, the critical role of IL-17A in the pathogenesis of psoriasis has been increasingly recognized, making it a key target in therapeutic research. In psoriasis, IL-17A secreted by Th17 cells acts as a major pro-inflammatory mediator, working in synergy with other cytokines such as TNF-α to drive inflammation (Chiricozzi et al., 2011).Compared with other biologic therapies, patients with scalp, nail, or genital involvement were significantly more likely to achieve clinical clearance by week 12 when treated with anti-IL-17A biologics (Piaserico et al., 2023). Secukinumab and ixekizumab are humanized monoclonal antibodies that specifically target the pro-inflammatory cytokine IL-17A (Pappka et al., 2013; Griffiths et al., 2015). Both have been approved in China for the treatment of moderate-to-severe plaque psoriasis in adults and were previously approved by the FDA and EMA for treating psoriatic arthritis and moderate-to-severe plaque psoriasis in individuals aged 6 years and older. Numerous studies, both within China and internationally, have demonstrated the strong therapeutic efficacy of IL-17A inhibitors, represented by secukinumab and ixekizumab, in treating psoriasis. A Phase III prospective, randomized, double-blind, placebo-controlled study conducted in 2017 evaluated the efficacy of secukinumab for treating moderate-to-severe scalp psoriasis (Bagel et al., 2017). After 12 weeks of treatment, 52.9% of patients in the secukinumab group achieved a 90% improvement in the Scalp Psoriasis Severity Index (PASI 90), compared to only 2% in the placebo group (p<0.001). Additionally, a significantly higher proportion of patients in the secukinumab group achieved complete scalp clearance (i.e., PASI 100) by week 12, 35.3% vs. 0% in the placebo group (p<0.001). By week 24, the proportion of patients achieving PASI 100 in the secukinumab group had increased to 47.1%. Additionally, a *post-hoc* analysis of a Phase II randomized, placebo-controlled trial and its open-label extension have confirmed the efficacy of ixekizumab in treating scalp psoriasis (Langley et al., 2015). The study included 105 patients with a baseline PASI score of 18.7. At week 12 of treatment, mean reductions in PASI scores were 75.3% (p=0.001), 83.7% (p=0.001), and 82.2% (p<0.001) for the 25mg, 75mg, and 150mg ixekizumab groups, respectively. Notably, by week 20, 58.3%, 66.7%, and 86.4% of patients in these respective dose groups achieved a PASI score of 0 (indicating complete lesion clearance), compared to only 10% of patients in the placebo group.

Research data have shown a close correlation between microbiota dysbiosis, both cutaneous and gut, and the pathogenesis and progression of psoriasis. Microbiota composition can vary based on the host’s geographic location, body surface area, age, presence of comorbidities, hygiene practices, medication use, and external conditions. On the skin, a dynamic interaction exists between the microbiota and host immune components, including Toll-like receptors, peptidoglycan recognition proteins, antimicrobial peptides, and cytokines (Fry et al., 2015). Studies have shown that microbial colonization33 \r \h t bacteria, fungi, viruses, mites, and their endosymbionts3 \r \ a significant role in skin diseases, particularly in scalp psoriasis. However, the importance of microbiota is often overlooked (Tatu and Cristea, 2017; Kubiak et al., 2018; Oh and Voigt, 2025). The scalp presents a particularly complex microecological environment due to its dense hair follicles and abundant sebaceous glands. Studies have demonstrated a strong correlation between the severity of scalp psoriasis and microecological imbalance (Choi et al., 2022). Psoriatic lesions are typically associated with an increased relative abundance of Corynebacterium, while the relative abundance of Cutibacterium acnes and certain Corynebacterium species is lower (Fyhrquist et al., 2019). Meanwhile, the gut, recognized as an important immune organ, exhibits significant immunomodulatory properties through its microbial composition and metabolites. Microbiome alterations associated with psoriasis can induce inflammatory responses by activating cytokines such as IL-23, IL-17, and IL-22, modulating interferon-gamma, and inhibiting the production of regulatory T cells. These immune dysregulations contribute to the excessive proliferation of keratinocytes. Multiple studies have demonstrated a strong link between gut dysbiosis and the pathogenesis of various diseases, including obesity, multiple sclerosis, inflammatory bowel disease, spondyloarthritis, and psoriasis (Dei-Cas et al., 2020). Studies have suggested that elevated IL-17 levels in lesional skin may serve as an underlying mechanism for head and neck dermatitis, a recognized adverse event associated with dupilumab therapy (Soria et al., 2019). This IL-17-mediated inflammatory response parallels findings in seborrheic dermatitis, where Malassezia-induced hydrolysis of free fatty acids similarly activates immune pathways involving IL-17 and IL-4 as key mediators (Adalsteinsson et al., 2020). These observations raise the possibility that microbial interactions may influence IL-17-dependent immune responses, particularly in head and neck dermatitis, suggesting a potential interplay between microbial communities, IL-17 signaling, and localized cutaneous inflammation in these anatomically complex regions.

However, the effects of IL-17A inhibitor therapy on the scalp and gut microbiota in patients with psoriasis remain unclear. To address this knowledge gap, this study aimed to explore the longitudinal changes in both the scalp and gut microbiota of patients with psoriasis before and after treatment with IL-17A inhibitors.

## 2 Methods and materials

### 2.1 Research participants

This study included a total of 15 patients diagnosed with moderate-to-severe psoriasis based on their Psoriasis Area and Severity Index (PASI) scores. All participants were hospitalized or attended the outpatient clinic at the Fifth People’s Hospital of Hainan Province between January 2024 and September 2024 and were receiving initial treatment with biologics (IL-17A inhibitors, such as secukinumab or ixekizumab). Additionally, 10 healthy individuals were included as controls. Inclusion criteria were as follows: ① Absence of concurrent seborrheic dermatitis on the scalp; ② No evidence of bacterial or fungal infection foci on the scalp; ③ No history of infectious diseases, autoimmune disorders, cardiovascular or cerebrovascular system diseases, malignant tumors, or other serious or progressive conditions; ④ Normal liver and kidney function; ⑤ No use of oral or topical glucocorticoids, anti-infective drugs (including antibacterial, antifungal, antituberculosis, antiparasitic drugs), retinoids, or probiotics within the past month. Participants who were pregnant or breastfeeding, along with those the researchers considered unsuitable for any other reason, were excluded from the study.

Scalp and fecal samples from participants in the psoriasis group were collected at baseline (M0), as well as at 4 weeks (M4) and 12 weeks (M12) following treatment with IL-17A inhibitors (ixekizumab or secukinumab). In contrast, for the healthy control group (C group), only baseline samples of scalp dandruff and feces were collected. Scalp dandruff samples were labeled as Group H, while fecal samples were labeled as Group F. IL-17A inhibitor treatments were strictly administered via subcutaneous injections according to the prescribed regimen (secukinumab: given at a dose of 300 mg at weeks 0, 1, 2, 3, and 4, followed by maintenance doses every 4 weeks; ixekizumab: administered as a 160 mg injection at week 0, followed by 80 mg doses at weeks 2, 4, 6, 8, and 12, and then continued every 4 weeks thereafter).

### 2.2 Sample collection

Sample collection time: 2 hours before IL-17A inhibitors treatment in the 0 th week, 4 th week and 12 th week respectively(S1).

Scalp dandruff specimens: A sterile cotton swab moistened with 10 mL of 0.9% sodium chloride injection was gently rolled and pressed over psoriatic scalp lesions for at least 30 s for collection. After collection, the swabs were placed into 2 mL centrifuge tubes containing lysis buffer and glycerol (as backup).

Fecal specimens: Fresh stool samples were collected, and approximately 2 g were obtained using a cotton swab. After collection, the swabs were placed into 2 mL centrifuge tubes containing lysis buffer and glycerol (as backup). All sample tubes were stored at -20°C within 2 hours of collection. For the C group, scalp samples were collected from the corresponding scalp regions as those in the psoriasis group using the same method.

### 2.3 DNA extraction, polymerase chain reaction amplification, and sequencing

Total genomic DNA from the skin microbiota of both the psoriasis group and healthy controls was extracted using a commercial DNA extraction kit (MJYH, Shanghai, China), according to the manufacturer’s instructions. Subsequently, the integrity of the extracted DNA was assessed, and its concentration and purity were determined. PCR amplification targeting the V3-V4 variable region of the 16S rRNA gene was performed using the extracted DNA as a template, with primers 338F (5’-ACTCCTACGGGAGGCAGCAG-3’) and 806R (5’-GGACTACHVGGGTWTCTAAT-3’), both containing barcode-specific sequences. The PCR products were recovered and purified, while the fragment sizes were analyzed. Quantification of the purified products was performed using Synergy HTX (Biotek, USA). These purified PCR products were then used to construct a library, and sequencing was conducted using the Illumina Nextseq2000 platform.

### 2.4 Sequencing analysis, gene function prediction, and statistical analysis

Quality control of paired-end raw sequencing reads was performed using the fastp software, followed by merging of the reads with the FLASH software. The optimized sequences obtained following quality control and merging were denoised using the DADA2 plugin within the Qiime2 pipeline. The sequences processed using DADA2 were termed Amplicon Sequence Variants (ASVs). Taxonomic classification of the ASVs was performed using the UNITE 9.0 gene database. Microbial functions were predicted using the PICRUSt2 algorithm, beginning with systematic annotation of functional genes based on the Kyoto Encyclopedia of Genes and Genomes (KEGG) Orthology. The annotated genes were then mapped to the KEGG PATHWAY database for pathway enrichment analysis, with comparisons made at the Level 2 pathway classification. Alpha diversity indices (ACE index, Chao index, sobs index, Shannon index, and Simpson index) were calculated using the Mothur software, and differences in α-diversity among groups were assessed using a one-way analysis of variance. For β-diversity analysis, Principal Coordinate Analysis (PCoA) based on the Bray-Curtis distance matrix was used to evaluate similarities in bacterial microbiota community structures across samples. The PCoA was combined with the Analysis of Similarities, a non-parametric statistical test, to analyze whether bacterial microbiota community structure differences among sample groups were statistically significant. Finally, the Kruskal-Wallis rank sum test was employed to identify species with significant differences among the groups.

## 3 Results

### 3.1 Clinical data and characteristics of participants

A total of 29 patients with psoriasis were initially included in this study. However, due to some patients missing scheduled biologic treatments or lacking complete clinical data, only 15 patients were included in the final analysis. From these 15 patients, 45 scalp dandruff samples and 45 fecal samples were collected at three time points (M0, M4, M12). Additionally, 10 scalp dandruff and 10 fecal samples were collected from the C group. This group included seven males and three females, aged 31–73 years, with a mean age of 51.50 ± 14.46 years. The psoriasis group comprised 12 males and three females, aged 25–74 years, with a mean age of 49.13 ± 14.37 years. Disease duration in the psoriasis group ranged from 0.34 to 30 years, with a mean duration of 8.75 ± 8.4 years. No statistically significant differences were observed in age (p=0.884) or sex (p=0.6695) between the control and psoriasis groups. Of the 15 patients with psoriasis, four were treated with secukinumab and 11 with ixekizumab. The PASI scores were 15.1–36.4 (mean 22.95 ± 7.13), 7.6–20.1 (mean 13.36 ± 4.12), and 0–7.9 (mean 3.99 ± 2.31) in the M0, M4, and M12 groups, respectively. Significant differences in PASI scores were observed across all three-time points (p<0.001). After 12 weeks of treatment, two patients achieved complete remission, 13 achieved significant remission, and two showed partial remission, resulting in a total response rate of 100% (**Supplementary Table 1**). We performed 16S rRNA sequencing analysis on scalp dandruff and fecal samples collected from patients with psoriasis at baseline and at 4 and 12 weeks post-treatment. Species rarefaction curves confirmed that the sequencing depth was sufficient to capture the majority of microbial species (S2, 3).

### 3.2 Changes in scalp and gut microecology after IL-17A inhibitor treatment

**Figure 1:**
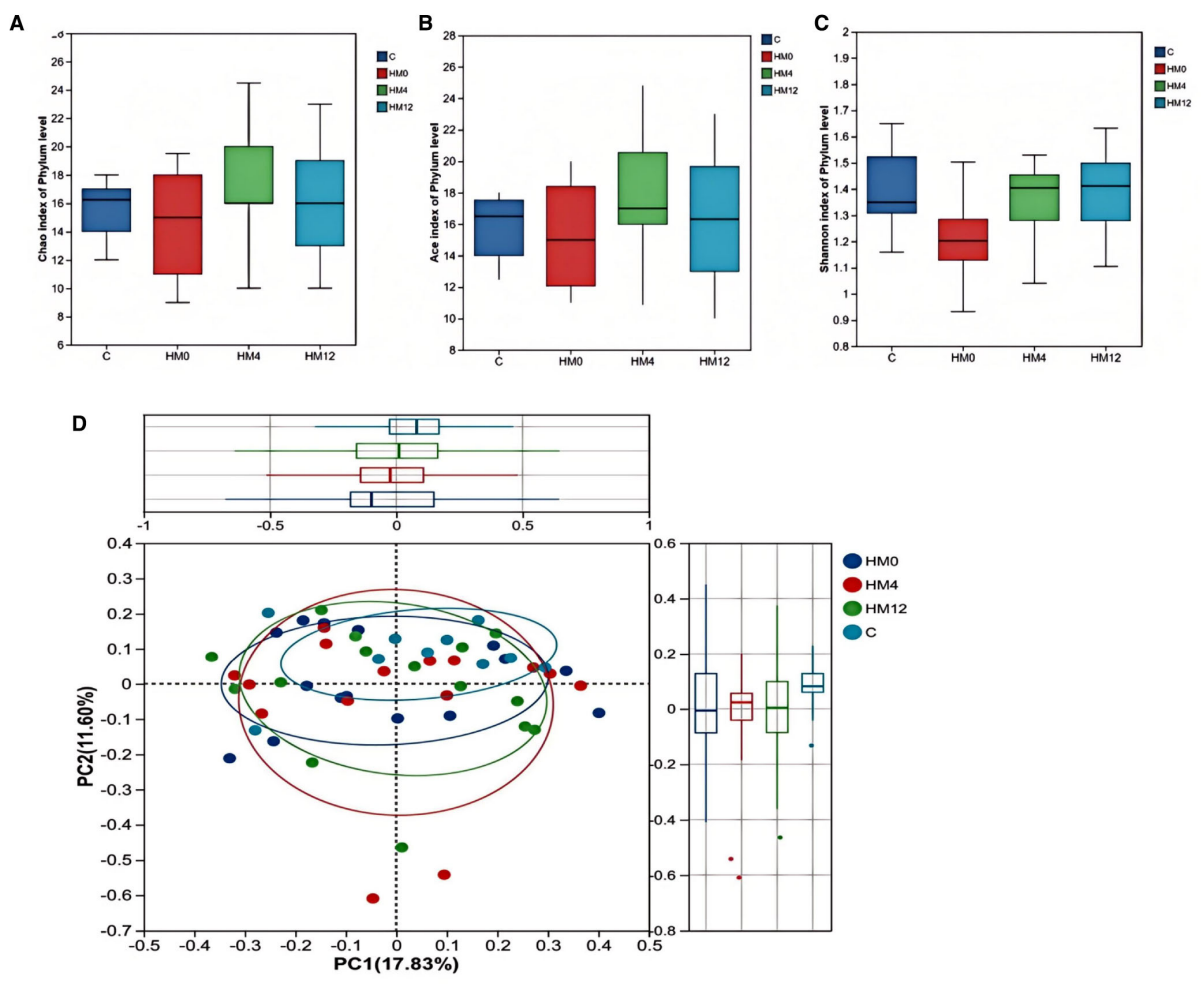
Bacterial community diversity in the healthy and psoriatic scalp. **(a)** Chao index. **(b)** Ace index. **(c)** Shannon index of each group in scalp samples. **(d)** Principal coordinate analysis (PCoA) of the microbial community structures based on weighted Bray-Curtis distance matrix for the first two principal axes. Each point on the PCoA plot represents a scalp microbiome ample. The first principal coordinate explains 17.83% of variation, and the second principal coordinate explains 11.60% of the variation.

**Figure 2:**
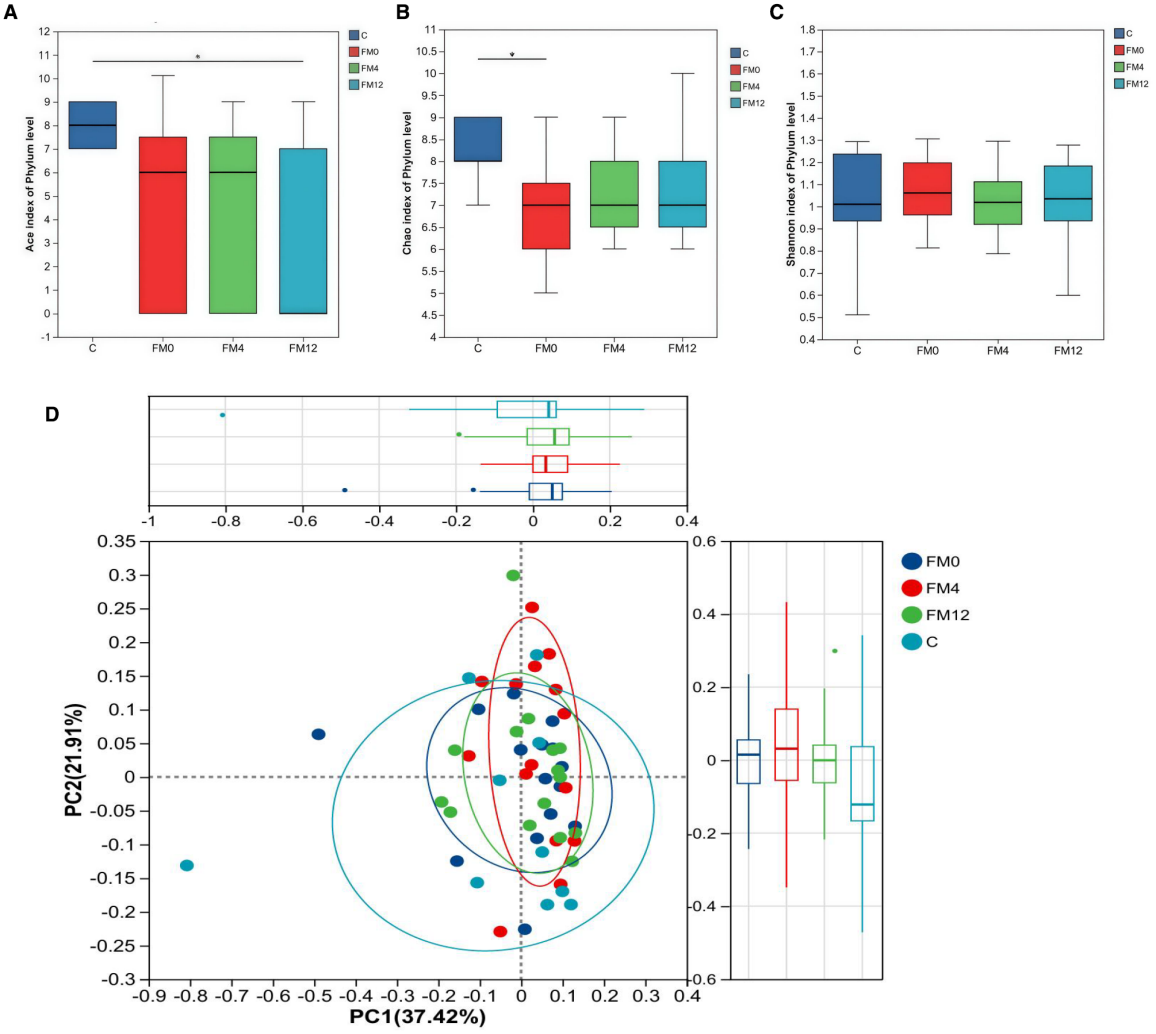
Bacterial community diversity in the healthy and psoriatic gut. **(a)** Ace index. **(b)** Chao index. **(c)** Shannon index of each group in fecal samples. **(d)** Principal coordinate analysis (PCoA) of the microbial community structures based on weighted Bray-Curtis distance matrix for the first two principal axes. Each point on the PCoA plot represents a fecal microbiome ample. The first principal coordinate explains 37.42% of variation, and the second principal coordinate explains 21.01% of the variation.

We examined the temporal effects of IL-17 inhibitors on scalp and gut microecology. Alpha diversity showed no statistically significant changes in the scalp microbiota before and after treatment. However, a trend was observed: the HM0 group showed decreased diversity compared to the C group, while diversity in the HM4 and HM12 groups increased and began to approach that of the C group. In fecal samples, significant differences in the ACE and Chao indices at the phylum level were observed between the control and FM0 groups (p<0.05), indicating a significant reduction in species richness in the psoriasis group compared to healthy controls. However, after IL-17A inhibitor treatment, no significant differences in species richness were observed among the psoriasis group (**Figures 1a–c**). Beta diversity analysis using PCoA based on the Bray-Curtis distance matrix showed distinct clustering patterns. In the scalp microbiota, samples from the C group were concentrated in the positive value range, while those from the HM0 group were concentrated in the negative value interval, indicating divergence and suggesting dysbiosis in the scalp microecology in the disease state. Following treatment, samples from the HM4 and HM12 groups tended toward the C group cluster, suggesting a partial restoration of the microbial community structure toward a healthy state, although not fully normalized (**Figure 1d**). At the phylum level, significant differences were observed in the alpha diversity Ace and Chao indices between Group C and FM0 in fecal samples (p < 0.05), whereas no statistically significant difference was found in the Shannon index (**Figures 2a–c**). PCoA also revealed separations among the C, FM0, FM4, and FM12 groups, indicating differences in microbial community composition. However, no clear directional trend toward recovery was observed (**Figure 2d**).

### 3.3 Changes in scalp and gut microbial community composition after IL-17A inhibitor treatment in patients with psoriasis

**Figure 3:**
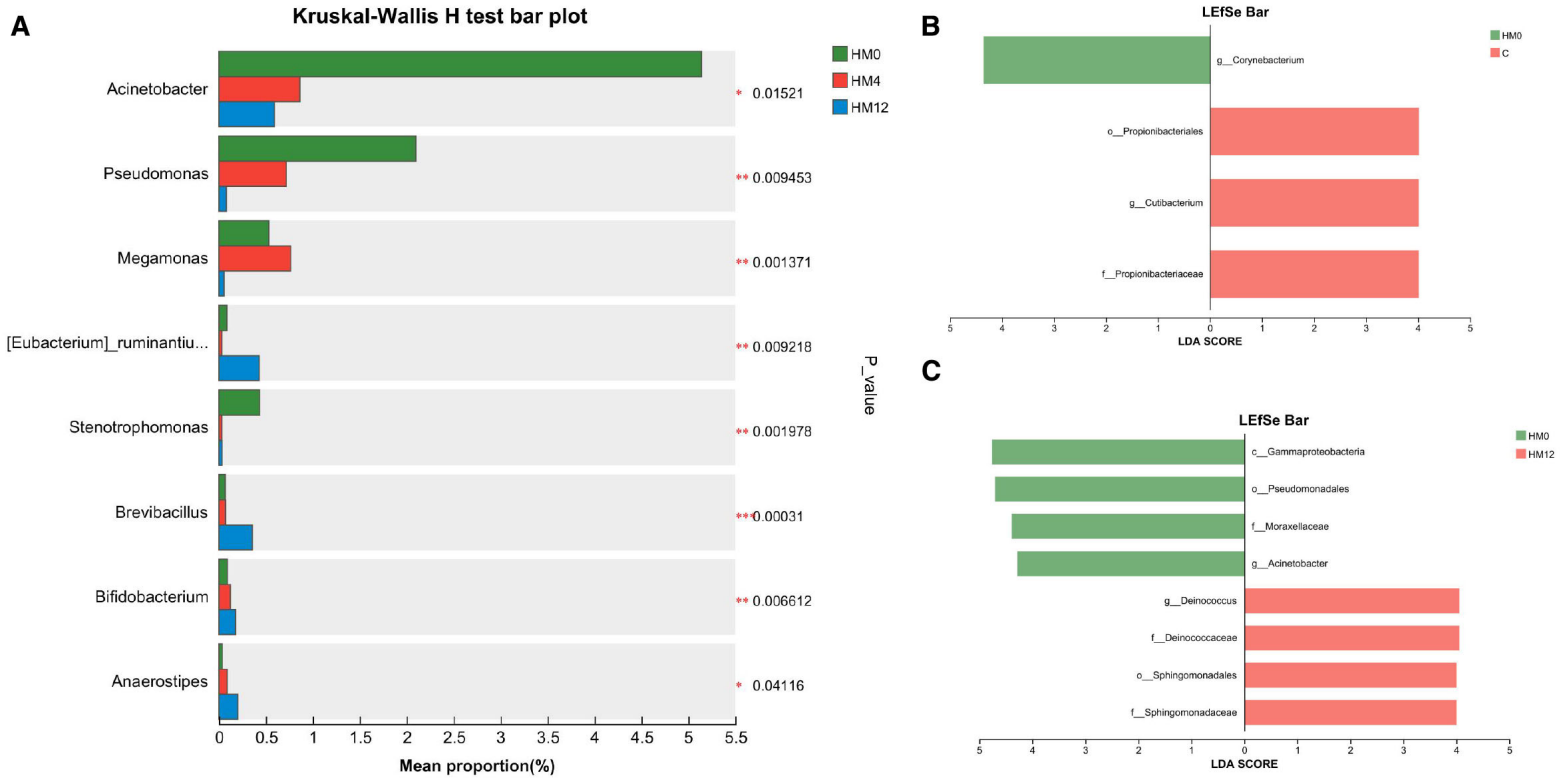
Microbial community analysis: **(a)** Differential species among HM0, HM4, and HM12 groups; **(b)** Bar plot of LDA value distribution between C and HM0 groups; **(c)** Bar plot of LDA value distribution in HM0 and HM12 groups.

To investigate the effects of IL-17A inhibitors on scalp and gut microbiota, we analyzed inter-group differences in relative abundance at various taxonomic levels (p<0.05) and found statistically significant inter-group differentially abundant species using LEfSe analysis based on ASV sets and microbial community abundance data. Following IL-17A inhibitor treatment, significant changes were observed in the scalp microbiome. At the genus level, the relative abundances of *Acinetobacter*, *Pseudomonas*, *Megamonas*, and *Stenotrophomonas* significantly decreased, while the relative abundances of *[Eubacterium]_ruminantium_group*, *Brevibacillus*, *Bifidobacterium*, and *Anaerostipes* significantly increased (p<0.05) (**Figure 3a**). LEfSe analysis found that, compared to the C group, the HM0 group showed enrichment of *Corynebacterium* at the genus level. In contrast, the C group was enriched in *Propionibacteriales* (order), *Propionibacteriaceae* (family), and *Cutibacterium* (genus) (**Figure 3b**). Longitudinal comparison between the HM0 and HM12 groups indicated that HM0 was characterized by an abundance of *Gammaproteobacteria* (class), *Pseudomonadales* (order), *Moraxellaceae* (family), and *Acinetobacter* (genus). Notably, *Propionibacterium*, which was significantly different between the HM0 and C groups, showed no significant changes post-treatment (**Figure 3c**).

**Figure 4:**
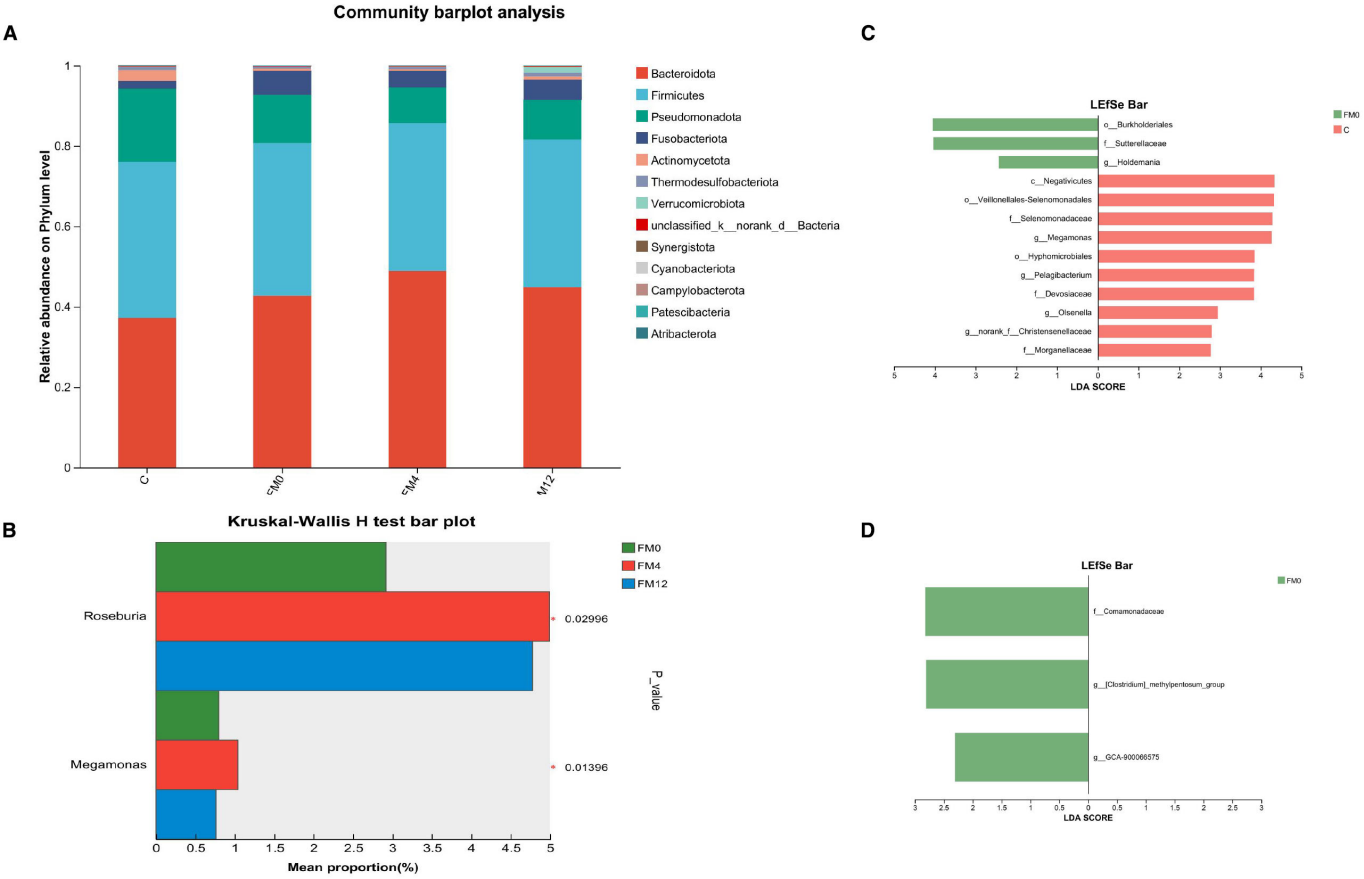
**(a)** Phylum-level microbial community bar plot of fecal samples. **(b)** Differentially abundant species among the FM0, FM4, and FM12 groups. **(c)** LEfSe analysis of variance in HM0 and C group. **(d)** LEfSe analysis of variance in HM0 and M12 group.

At the phylum level, the dominant bacterial phyla in the gut microbiota were *Bacteroidota*, *Firmicutes*, *Pseudomonadota*, *Fusobacteriota*, and *Actinomycetota* (**Figure 4a**). IL-17A inhibitor treatment led to increased relative abundances of *Roseburia* and *Megamonas* at the genus level (**Figure 4b**). LEfSe analysis (LDA threshold > 2.0) identified distinct biomarkers between groups: FM0 showed enrichment in Burkholderiales (order), Sutterella (family), and Holdemania (genus), while Group C was characterized by Negativicutes (class) and Hyphomicrobiales (order),et al (**Figure 4c**). LEfSe analysis further showed that in the FM0 group, the differentially abundant species between FM0 and FM12 groups were *Comamonadaceae*, *[Clostridium]_methylpentosum_group*, and *GCA-900066575* (NCBI database classification) (**Figure 4d**).

### 3.4 Functional prediction of scalp and gut microbial communities after IL-17A inhibitor treatment in patients with psoriasis

**Figure 5:**
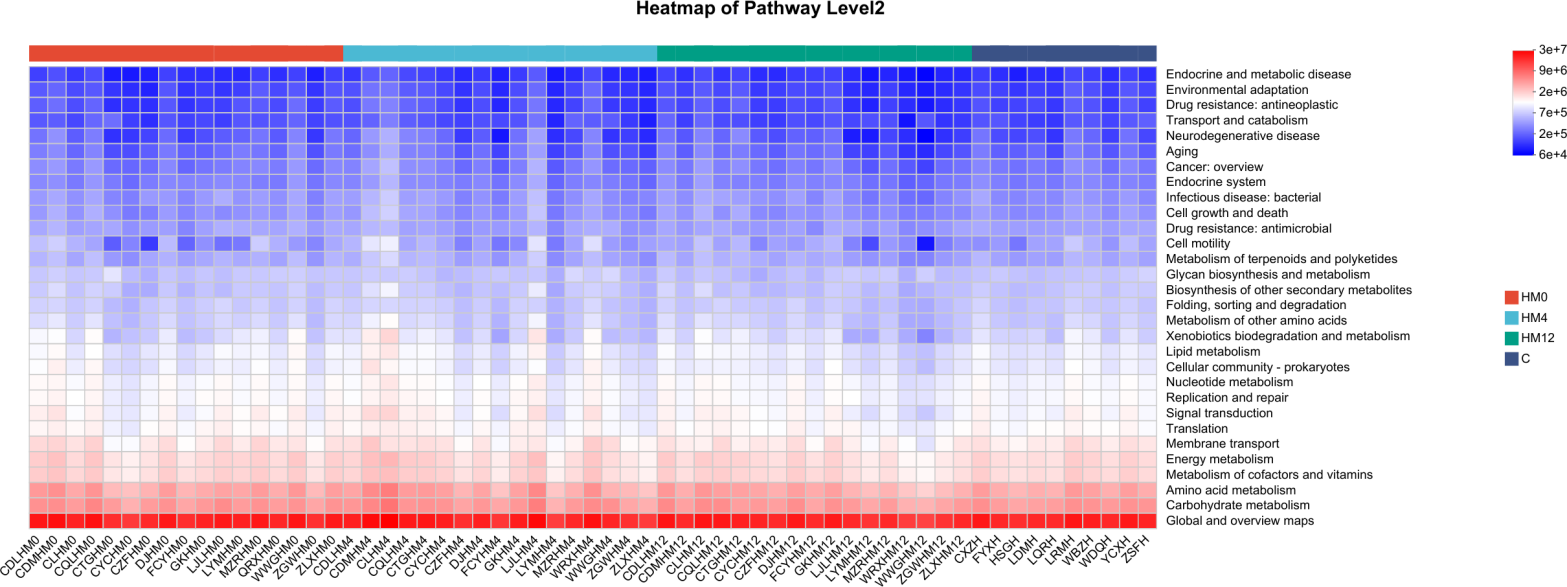
Functional analysis heatmap of KEGG metabolic pathways in scalp samples.

Functional prediction analysis revealed notable shifts in microbial metabolic pathways following IL-17A inhibitor treatment. In the scalp microbiota, compared to the C group, the HM0 showed enrichment in pathways related to “Endocrine and metabolic diseases” and “Drug resistance: antineoplastic”. After treatment, the HM4 and HM12 groups exhibited increased abundance in pathways associated with “Energy metabolism” and “Amino acid metabolism,” while the abundance of “Drug resistance” pathways decreased (**Figure 5**).

**Figure 6:**
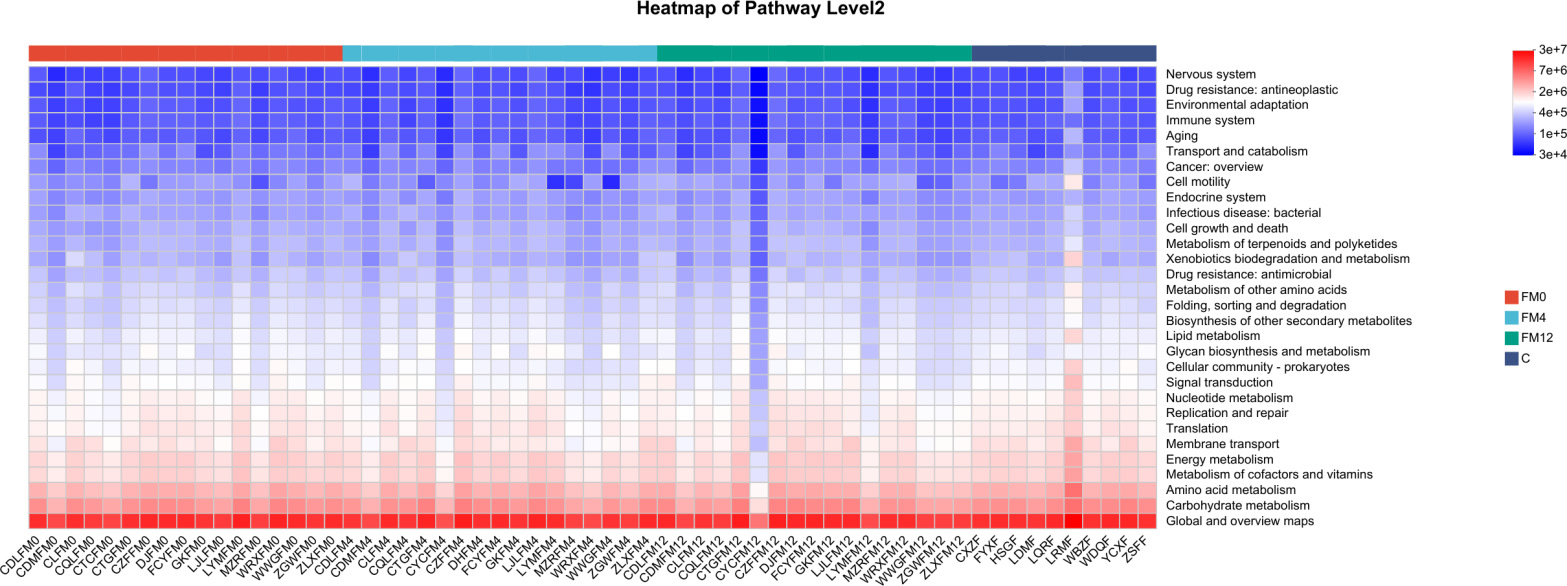
Functional analysis heatmap of KEGG metabolic pathways in fecal samples.

In the gut microbiota, a comparison between the FM0/and C groups indicated significant enrichment of pathways involved in “Drug resistance” and “Infectious diseases” in the FM0 group. Longitudinal analysis across the FM0, FM4, and FM12 groups showed a progressive increase in the “Environmental adaptation” pathway, along with a decline in both “Cell growth and death” and “Global and overview maps” pathway abundances (**Figure 6**).

## 4 Discussion

With the growing understanding of psoriasis pathogenesis in recent years, biologics have increasingly been used for targeted immunotherapy. These include tumor necrosis factor-alpha (TNF-α) inhibitors, interleukin (IL)-17A inhibitors, IL-12/23 inhibitors, and IL-23 inhibitors, which have gained widespread attention due to their high efficacy and relatively few adverse effects (Memariani and Memariani, 2025). In this study, all patients treated with IL-17A inhibitors achieved a positive clinical response. By 12 weeks (M12), two patients achieved complete remission, 13 achieved significant remission, and two achieved partial remission, resulting in a total response rate of 100%. These findings are consistent with previous real-world studies conducted in China (Ying et al., 2022; Zhou et al., 2024). In contrast, a cohort study evaluating the effectiveness and durability of oral treatments (acitretin, cyclosporine, methotrexate, and fumarate) in patients with moderate-to-severe psoriasis showed that approximately 21% (118/563) of patients treated with acitretin capsules achieved a Psoriasis Area and Severity Index (PASI) ≤2 (corresponding to PASI 90), with a median onset time of 10.4 months (interquartile range: 5.7–16.5). Compared to these oral therapies, IL-17A inhibitors demonstrated a more rapid onset and superior efficacy (Alabas et al., 2023).

In this study, 16S rDNA high-throughput sequencing was employed to analyze the scalp and gut microbiota of 15 patients with psoriasis vulgaris before and after treatment with IL-17A inhibitors. Microbial diversity and community structure were compared among patients with psoriasis at baseline, 12 weeks post-treatment, and healthy controls. The aim was to explore characteristic changes in the scalp and gut microbiota during the recovery process following IL-17A inhibitor therapy in patients with psoriasis.

In this study, no significant differences were observed in scalp microecological α-diversity and β-diversity between patients with psoriasis and healthy controls. However, diversity analysis showed a clear separation between the C and HM0 groups, with the HM4 and HM12 groups gradually shifting closer to the C group. This suggests that IL-17A inhibitor treatment may promote the restoration of species richness, diversity, and microbial community structure in the scalp microecology of patients with psoriasis toward a healthy state. These findings differ from those reported by Jin-Young Choi and Melek Aslan et al (Choi et al., 2022; Kayıran et al., 2022),possibly due to differences in sampling methods or specimen size. Further studies and meta-analyses involving larger populations are needed to clarify this discrepancy. Regarding gut microecology, α-diversity analysis showed significant differences in microbial richness between the psoriasis and healthy control groups (p<0.05). This result is consistent with the findings of Codoñer et al. (2018) who reported significantly altered α-diversity in patients with psoriasis using 16S rRNA high-throughput sequencing. However, studies conducted by Tan et al. (2018) and Sikora et al. (2020) on populations from the same region showed no significant differences in α-diversity between patients with psoriasis and healthy controls. Notably, discrepancies among study findings may stem from differences in experimental design. For instance, Tan et al.’s study did not account for potential confounding factors such as prior drug use (e.g., antibiotics) or comorbid metabolic conditions (e.g., diabetes), which could affect the accuracy of gut microbiota analyses. β-diversity analysis in the present study showed distinct differences in bacterial community composition across groups, with the microbial community structure post-treatment (M4/M12) diverging from baseline (M0), although it did not fully restore to a healthy state. These findings indicate that IL-17A inhibitors may affect gut microecology by modulating the abundance or metabolic pathways of specific microbiota.

To further explore microecological changes before and after IL-17A inhibitor treatment, a longitudinal multi-group analysis was conducted comparing the HM0, HM4, and HM12 groups. The results showed that, in the psoriasis group, the relative abundance of *Proteobacteria* significantly decreased, while *Firmicutes*, *Bacteroidetes*, and *Actinobacteria* increased in the HM12 group compared to the HM0 and HM4 groups. At the genus level, the dominant bacterial genera included *Halomonas*, *Staphylococcus*, *Bacteroides*, and *Corynebacterium*. Notably, following IL-17A inhibitor treatment, the relative abundances of pathogenic bacteria *Acinetobacter* and *Pseudomonas* decreased. In psoriatic skin, human β-defensin-2 is highly expressed in the stratum corneum, and its serum levels have been shown to correlate with disease severity (Hollox et al., 2008). *Pseudomonas* is a potent inducer of human β-defensin-2 in both skin and lung epithelial tissues (Harder et al., 2000; Harder et al., 1997). Beyond its antimicrobial properties, this peptide may play a crucial role in recruiting Th17/22 cells, contributing to the pathogenesis of psoriasis (Furue et al., 2020). Although the relationship between the skin microbiome and human β-defensin-2 is not yet fully understood, commensal bacteria may interact with this antimicrobial peptide and influence its activities. Moreover, oral supplementation with *Bifidobacterium* has been shown to alleviate psoriasis in a dose-dependent manner by restoring the microbiota, promoting bile acid production, regulating the FXR/NF-κB pathway, reducing pro-inflammatory cytokines, regulating keratinocytes, and maintaining epidermal barrier function (Xinqi et al., 2023). In summary, following IL-17A inhibitor treatment, the abundance of the harmful bacterium *Pseudomonas* in the scalp decreased, while the abundance of the beneficial bacterium *Bifidobacterium* increased, potentially influencing the severity of psoriasis. Notably, in our study, LEfSe analysis showed a decrease in *Propionibacterium* (phylum *Actinobacteria*) in psoriatic scalp lesions, consistent with findings reported by Quan et al (Quan et al., 2020)and Fyhrquistet al (Fyhrquist et al., 2019). *Propionibacterium* expresses antimicrobial activity by producing molecules such as short-chain fatty acids (SCFAs) and thiopeptides, which exhibit inhibitory effects. An imbalance in immunomodulatory microbiota such as *Propionibacterium* may promote colonization by pathogens such as *Staphylococcus aureus*, leading to reduced microbial community stability and decreased microbial diversity. This shift may induce TH17 cell activation and exacerbate skin inflammation through the Th17 axis (Chang et al., 2018). However, in the present study, no significant increase in *Propionibacterium* abundance was observed after IL-17A inhibitor treatment, and the mechanisms underlying this observation require further exploration.

In the analysis of gut microbial communities, *Bacteroidota* and *Firmicutes* were identified as the dominant phyla. Although we observed dynamic changes in the Firmicutes-to-Bacteroidetes (F/B) ratio across different groups, no significant correlation with clinical disease progression was found. Longitudinal comparisons within the psoriasis group before and after treatment, along with multi-group differential species analysis, revealed increased abundances of *Roseburia* and *Megamonas* following IL-17A inhibitor treatment, both of which belong to the *Firmicutes* phylum. Additionally, community composition bar charts showed a notable increase in *Bacteroidetes*. Previous studies on biologic treatments for inflammatory bowel disease have also reported changes in the gut microbiota, including increased levels of SCFA-producing bacteria, which help correct gut microbial dysbiosis and are considered beneficial commensals (Seong et al., 2020; Franzin et al., 2021). Both *Firmicutes* and *Bacteroidetes* are known producers of SCFAs, including acetate, propionate, and butyrate. *Bacteroidetes* primarily produce acetate and propionate, while *Firmicutes* are the main producers of butyrate (den Besten et al., 2013). Butyrate plays a critical role in maintaining the integrity of the intestinal epithelial barrier and exhibits strong anti-inflammatory properties. In addition, butyrate can inhibit oxidative stress and help regulate the balance between Th17 and Treg lymphocytes (Zeng et al., 2017; Myers et al., 2019; Polak et al., 2021). *Bacteroides* are among the most prevalent bacterial genera in the human gut (Sears, 2005), and their role in immune regulation has been demonstrated in animal studies. Supplementation with *Bacteroides* has been shown to alleviate colitis symptoms in mice and reduce the expression of pro-inflammatory cytokines such as TNF-α, IL-1β, and IL-6 in the colon (Liu et al., 2022). Furthermore, mice treated with oral *Bacteroides* exhibited inhibition of both systemic and intestinal immune responses (Wexler and Goodman, 2017; Yoshida et al., 2018).In this study, we observed increased relative abundances of *Firmicutes* and *Bacteroidetes* following IL-17A inhibitor treatment. These findings suggest that IL-17 inhibitors may exert anti-inflammatory effects in psoriasis by regulating gut dysbiosis through metabolites and intestinal immunity modulation.

Additionally, we explored the functional changes in the scalp and gut microbiota associated with IL-17A inhibitor treatment. Using the KEGG metabolic pathway analysis, we predicted the metabolic pathways with differential abundances across groups. In scalp microbiota, the “Endocrine and metabolic disease” and “Drug resistance: antineoplastic” pathways were significantly enriched in the HM0 group compared to the C group, potentially indicating metabolic dysfunction or enrichment of drug resistance-related genes in the scalp microbiota of patients with psoriasis, which may be linked to the disease state. After treatment (HM4 and HM12), the abundance of “Energy metabolism” and “Amino acid metabolism” pathways increased, potentially indicating improved microbial metabolic function. These changes reflect a partial restoration of gut microbial metabolic activity and support for host energy balance. The decrease in the abundance of the “Drug resistance” pathway suggests that IL-17A inhibitor treatment may reduce microbial drug resistance pressure, indicating improved microbial metabolic function, reduced drug resistance, and enhanced scalp microecological health. In the gut microbiota, compared to the C group, the “Drug resistance” and “Infectious disease” pathways were significantly elevated in the FM0 group, suggesting that baseline gut microbiota in patients with psoriasis may exhibit characteristics of drug resistance and pro-inflammatory activity. Longitudinal comparisons among the FM0, FM4, and FM12 groups showed a gradual increase in the “Environmental adaptation” pathway, reflecting enhanced microbial adaptation to treatment. Additionally, a decrease in the “Cell growth and death” pathway was observed, which may be associated with reduced inflammation, such as inhibition of apoptotic signaling. The reduction in the “Global and overview maps” pathway indicates the stabilization of overall microbial metabolic activity. Collectively, these changes in metabolic pathways suggest that IL-17A inhibitor treatment may promote microbial adaptation, inhibit inflammation-related processes, and support gut microecological health by modulating microbial immune and metabolic functions.

This study applied strict inclusion criteria regarding age, sex, underlying conditions, and the absence of prior treatments affecting the scalp or gut microbiota. Although individuals with unusual shampooing habits were excluded, and participants were instructed to avoid yogurt, probiotics, and similar foods during the study period, individual variations remained. These included differences in shampoo product selection, frequency of use, application methods, and daily dietary habits, which were difficult to standardize. Future work should therefore expand the sample size, prolong follow-up beyond 12 weeks, and incorporate longitudinal control sampling or animal models to validate functional predictions and disentangle treatment effects from background fluctuations. Secondly, although we revealed the influence of IL-17A inhibitors on the prediction of the structure and function of the microbiome, this study lacked quantitative analysis of systemic inflammatory markers (such as serum cytokines) and microbial functional products (such as short-chain fatty acids). Moreover, the function prediction of this study is based on the bioinformatics inference (PICRUSt2) of 16S rRNA sequencing data, which has not been verified by metagenomics, transcriptomics or metabonomics. Therefore, the changes in metabolic pathways of microbial communities reported by us should be regarded as preliminary and hypothesis-driven findings, and these predicted results provide valuable directions for further mechanism research. In the future, we will adopt multi-omics methods (such as transcriptomics and metabonomics) and serum cytokine analysis, which will help to establish the dynamic relationship between immune indexes, microbial changes and clinical phenotypes, so as to understand the therapeutic mechanism of IL-17A inhibitors more comprehensively.

## 5 Conclusion

IL-17A inhibitors demonstrate strong efficacy in treating psoriasis vulgaris. Although the scalp microecological diversity in patients with psoriasis does not differ significantly from that of healthy individuals, IL-17A inhibitor therapy improves the structure and composition of scalp microbiota, leading to a community profile that more closely resembles that of healthy controls, marked by reduced harmful bacteria, and increased beneficial ones. In contrast, gut microbial diversity in patients with psoriasis is significantly lower than in healthy individuals but also improves following IL-17A inhibitor treatment. The treatment leads to an increase in the abundance of *Firmicutes* and *Bacteroidetes*, although the F/B ratio remains unchanged. IL-17A inhibitors may reshape both scalp and gut microecology through immunomodulation, particularly by targeting the Th17 pathway and through changes in microbial metabolites such as SCFAs. Under IL-17A inhibition, patients exhibit marked microbial community remodeling, which may be closely linked to the pathogenesis of psoriasis. These findings underscore the importance of further exploration into the interaction between microbial homeostasis and immune regulation. A deeper understanding of this relationship could clarify the key role of the microbiota in psoriasis pathogenesis as well as identify novel biological targets for innovative therapeutic strategies.

## Data Availability

The raw data supporting the conclusions of this article will be made available by the authors, without undue reservation.
