# Supplementary material for: The impact of IL-17A inhibitors on scalp and gut microbiota in psoriasis
Source: Front Cell Infect Microbiol. 2025 Oct 6;15:1623003. doi: 10.3389/fcimb.2025.1623003 (PMC12536032; doi:10.3389/fcimb.2025.1623003)
Supplement: Supplementary file 1 [file Table1.docx]

# 附录

Table1 Clinical information of subjects in the psoriasis group

|  | Age | sexuality | course（year） | PASI（M0） | PASI（M4） | PASI（M12） |
| --- | --- | --- | --- | --- | --- | --- |
| CTG | 74 | 男 | 10 | 18.7 | 14.3 | 4.3 |
| MZR | 33 | 女 | 0.58 | 34.6 | 19.3 | 6.7 |
| CDM | 58 | 男 | 10 | 25.2 | 16.4 | 5.2 |
| CZF | 33 | 男 | 0.34 | 16.4 | 9.4 | 1.9 |
| WRX | 36 | 男 | 13 | 21.5 | 10.5 | 4.5 |
| FCY | 56 | 男 | 6 | 18.3 | 10.4 | 0 |
| CL | 25 | 女 | 5 | 27.4 | 14.7 | 5.4 |
| DJ | 42 | 男 | 0.58 | 22.1 | 13.8 | 5.6 |
| CDL | 48 | 男 | 0.5 | 33.6 | 19.6 | 4.8 |
| GK | 35 | 男 | 10 | 36.4 | 20.1 | 7.9 |
| CYC | 61 | 男 | 10.2 | 23.3 | 13.9 | 4.6 |
| ZGW | 67 | 男 | 15 | 25.2 | 16.4 | 3.7 |
| LJL | 49 | 男 | 22.5 | 15.2 | 7.6 | 3.5 |
| LYM | 52 | 男 | 1.3 | 15.1 | 8.3 | 0 |
| ZLX | 49 | 女 | 30 | 16.8 | 10.3 | 4.3 |


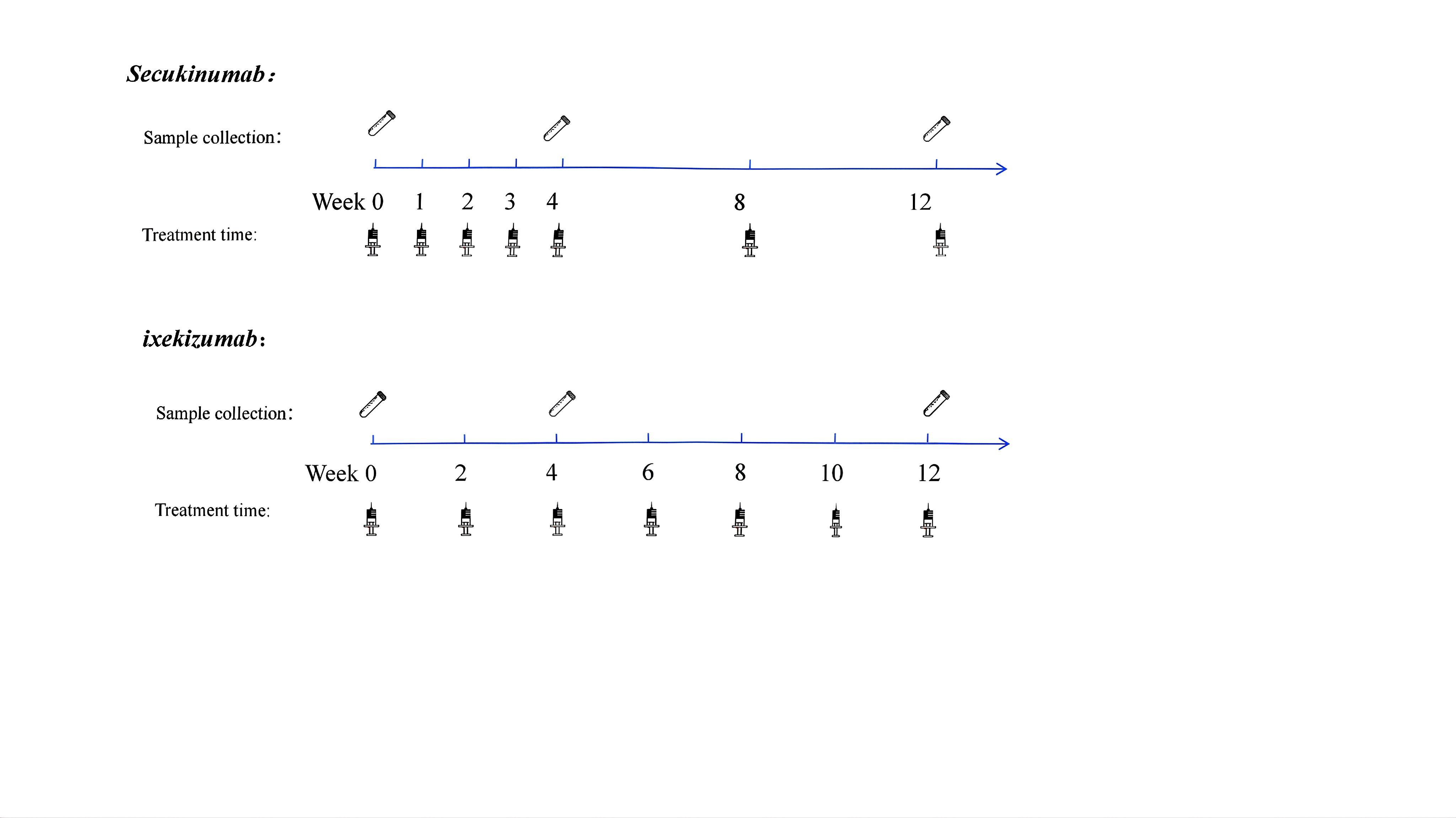


S1：Sample collection timeline


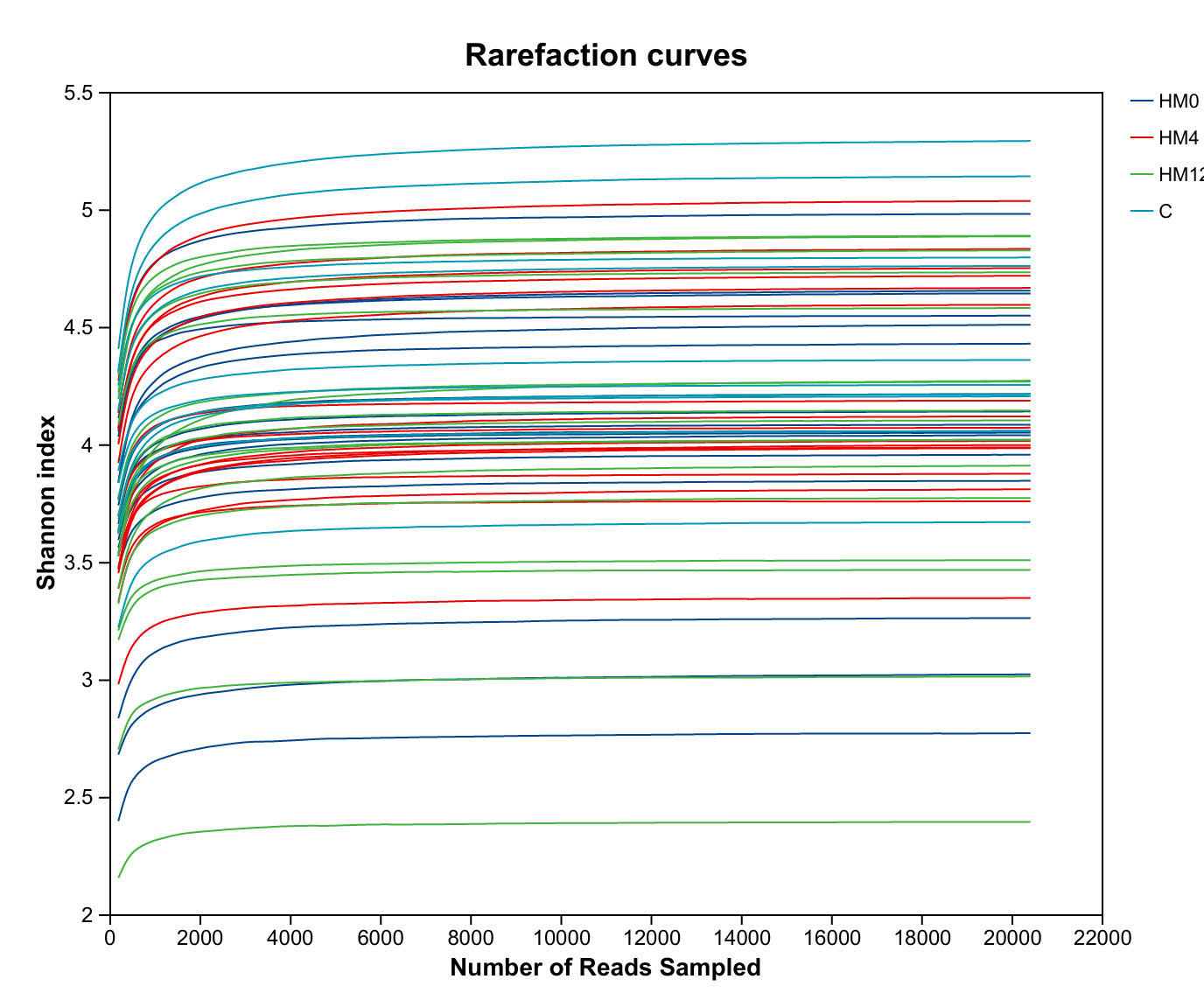


S2. The rarefaction curve for scalp samples based on the ASV-level Shannon index


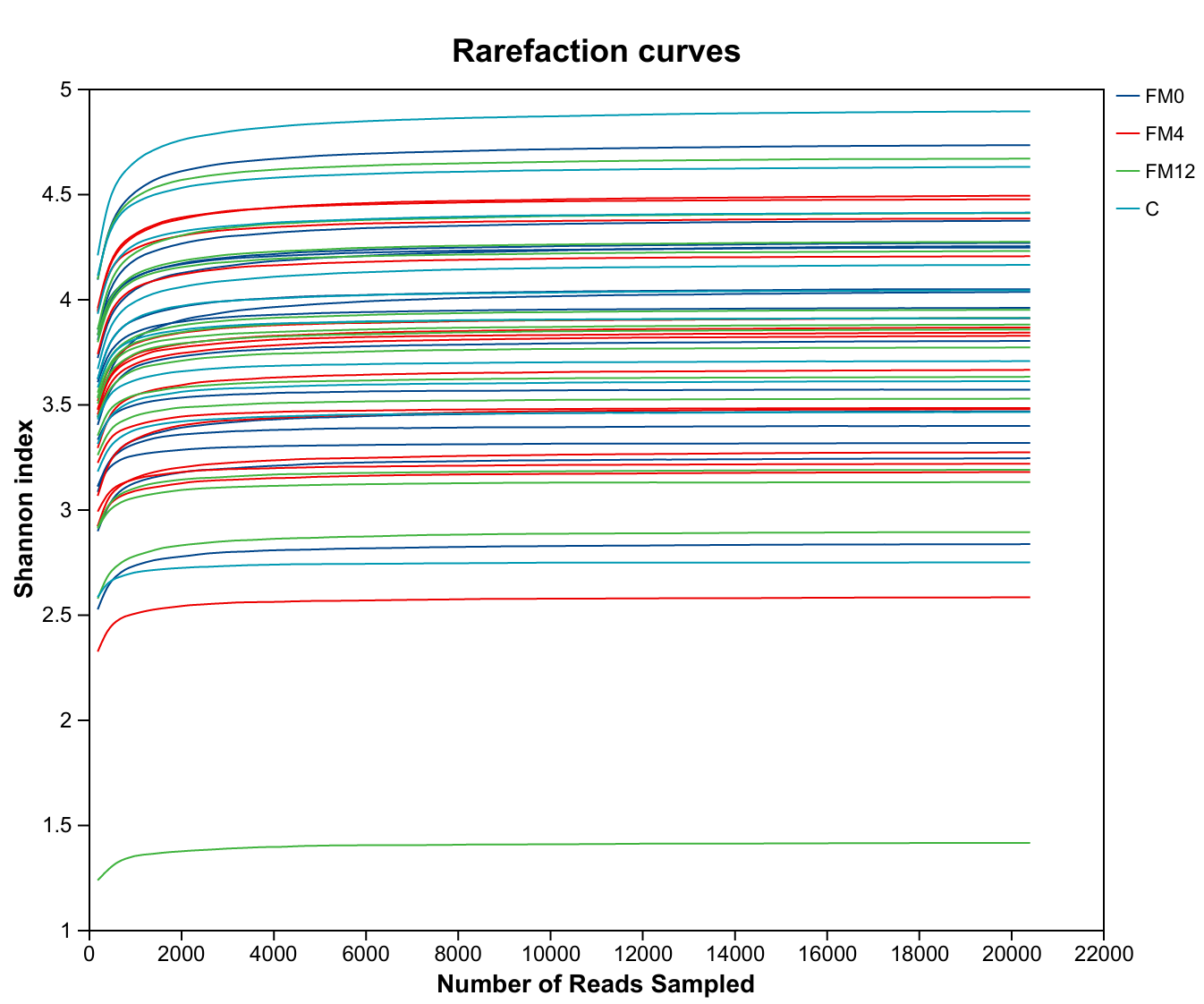


S3. The rarefaction curve for fecal samples based on the ASV-level Shannon index
